# Case series of neonatal lamb diarrhea syndrome treated with an evidence-based protocol to reduce antibacterial use

**DOI:** 10.1007/s11259-026-11413-y

**Published:** 2026-07-20

**Authors:** Aikaterini T. Pazarakioti, Labrini V. Athanasiou, Serafeim C. Chaintoutis, Panagiotis D. Katsoulos

**Affiliations:** 1 Clinic of Farm Animals, School of Veterinary Medicine, Aristotle University of Thessaloniki, Thessaloniki, Greece; 2 Clinic of Medicine, Faculty of Veterinary Medicine, University of Thessaly, Karditsa, Greece; 3 Diagnostic Laboratory, School of Veterinary Medicine, Aristotle University of Thessaloniki, Thessaloniki, Greece

**Keywords:** Neonatal enteritis, Ovine neonatology, Oral rehydration therapy, Bacteremia, Antimicrobial stewardship

## Abstract

Neonatal diarrhea in lambs remains a major cause of morbidity and mortality worldwide, yet standardized evidence-based treatment protocols for this species are limited. This prospective case series describes the field outcomes of a practical treatment protocol based on early intervention, oral rehydration, and targeted antibacterial use on a commercial Lacaune dairy sheep farm. The protocol was applied to 103 diarrheic lambs out of 133 born over a 60-day period. Daily clinical assessment included fecal scoring, evaluation of bacteremia signs, hydration status, suckling reflex, and abomasal palpation. All lambs continued voluntary suckling and received oral electrolyte solution daily until recovery. Antibacterials and NSAIDs were administered when at least two bacteremia signs were present, while isolated pyrexia was treated with NSAIDs alone and prolonged diarrhea (> 5 days) with antibacterials only. Mean duration of diarrhea was 4.0 days. Tested diarrheic lambs showed co-infection with rotavirus and *Cryptosporidium spp*. Bacteremia-compatible signs were observed in 25% of cases, and dehydration in 19%. Approximately 15% of lambs initially presented with isolated pyrexia before developing additional signs compatible with bacteremia, highlighting the importance of close clinical monitoring. Plasma glucose, sodium and potassium concentrations remained statistically stable. There was a 2.9% mortality rate, with all deaths attributed to *Escherichia coli* bacteremia. Antibacterials were required in only 34% of lambs, indicating that clinical selection criteria may help reduce unnecessary antibiotic exposure. Under field conditions, this protocol was associated with favorable clinical outcomes and supports early intervention, close monitoring, and selective antibacterial use in neonatal lamb diarrhea.

## Background

In sheep production systems, neonatal lamb health is a major factor in determining welfare, productivity, and overall sustainability of the herd. Diseases affecting lambs in the early stages of life are responsible for a significant proportion of lamb losses prior to weaning, with gastrointestinal disorders being among the most prevalent entities (Bangar et al. 2022). Neonatal diarrhea syndrome (NDS) is one of the most important challenges during the first few weeks of life and is a major cause of morbidity and mortality in lambs worldwide (Sharif et al. 2005; Hadgu et al. 2021; Fesseha et al. 2023). Beyond its direct impact on mortality, NDS exerts important sublethal effects on surviving lambs. It has been demonstrated that lambs recovering from neonatal diarrhea may exhibit delayed growth rates and reduced feed efficiency, which can persist well beyond the acute disease phase and lower lifetime productivity (Jacobson et al. 2016; Zhong et al. 2022). These outcomes negatively affect flock performance, increase replacement rates, and impose additional economic and management burdens on sheep enterprises. As a result, effective prevention and management of NDS remain a priority in modern sheep production systems.

Neonatal diarrhea syndrome is a disorder of multifactorial origin, arising from complex interactions between infectious agents, environmental factors, and the immunological status of the neonate (Martella et al. 2015). Enterotoxigenic *Escherichia coli*, rotavirus, coronavirus, and *Cryptosporidium* spp. have been identified as the primary pathogens implicated in the syndrome (Sargison 2004; Dahmani et al. 2020), with mixed infections being common and often associated with increased disease severity (Grünberg 2021; Delling and Daugschies 2022). In field conditions, NDS is most frequently attributed to *Cryptosporidium* spp. and rotaviruses, which may occur either as single infections or, more commonly, in combination (Delling and Daugschies 2022). Although the infectious causes of NDS have been extensively described, evidence-based treatment approaches for affected lambs remain poorly defined.

Pathogen-induced enteric damage activates both trigger secretory and malabsorptive mechanisms, rapidly leading to dehydration, electrolyte imbalances, metabolic acidosis, and hypoglycemia in affected lambs (Heller and Chigerwe 2018). Moreover, beyond the primary intestinal disease, a proportion of lambs may develop secondary intestinal bacterial overgrowth and subsequent bacteremia. While this complication has been well documented in calves, with bacteremia reported in approximately 30% of diarrheic cases (Fecteau et al. 1997b; Lofstedt et al. 1999), the incidence of bacteremia in lambs with neonatal diarrhea has not yet been clearly defined.

In many sheep production systems, treatment decisions for neonatal diarrhea are predominantly empirical, based on long-standing practices, convenience, or concern about potential disease progression rather than on standardized, evidence-based protocols. Unlike calves, for which structured and evidence-based therapeutic approaches to neonatal diarrhea are well described, comparable protocols tailored to neonatal lambs are largely lacking. As a result, clinical outcomes may be inconsistent, and affected lambs may receive unnecessary or inappropriate treatment. Of particular concern is the frequent and often unjustified use of antibacterial agents in lambs with diarrhea. Surveys of small ruminant farms have documented that antibacterials are routinely administered for diarrheic lambs on a large proportion of farms (Lianou and Fthenakis 2022), often without clear clinical justification or targeted diagnostic evidence to support their use, while supportive therapies such as electrolyte administration are inconsistently applied. This practice increases treatment costs and contributes to the development and selection of antibacterial resistance in sheep populations, a recognized concern in small ruminant veterinary practice (Scott and Menzies 2011). Responsible antibacterial use requires treatment to be guided by clear clinical criteria and evidence of systemic involvement rather than empirical administration at the onset of diarrhea.

This issue is particularly relevant within the broader One Health framework, which emphasizes prudent and targeted antibacterial use in veterinary medicine. In this context, reducing unnecessary antibacterial administration is aligned with the World Health Organization Global Action Plan on Antimicrobial Resistance, which identifies optimization of antimicrobial use in human and animal health as a core objective (World Health Organization 2015). It is also consistent with the current European Union regulatory framework for veterinary medicinal products established by Regulation (EU) 2019/6 (European Parliament and the Council of the European Union 2019), as well as with broader European policy initiatives aimed at reducing overall sales of antimicrobials for farmed animals and aquaculture by 50% by 2030 (European Commission 2020). In addition, the European Medicines Agency now centralizes and publishes annual ESUAvet surveillance data to monitor progress towards prudent antimicrobial use in animals across Europe (European Medicines Agency 2025).

Given the clinical importance of NDS and the current lack of standardized therapeutic guidance for lambs, there is a clear need for practical evidence-based treatment strategies under field conditions. Therefore, the objective of the present prospective clinical study was to propose and evaluate an evidence-based treatment protocol for neonatal lamb diarrhea syndrome based on early supportive care, systematic clinical monitoring, and selective antibacterial administration, with the aim of improving outcomes while reducing unnecessary antibiotic use.

## Case presentation

### Case history

**Fig. 1:**
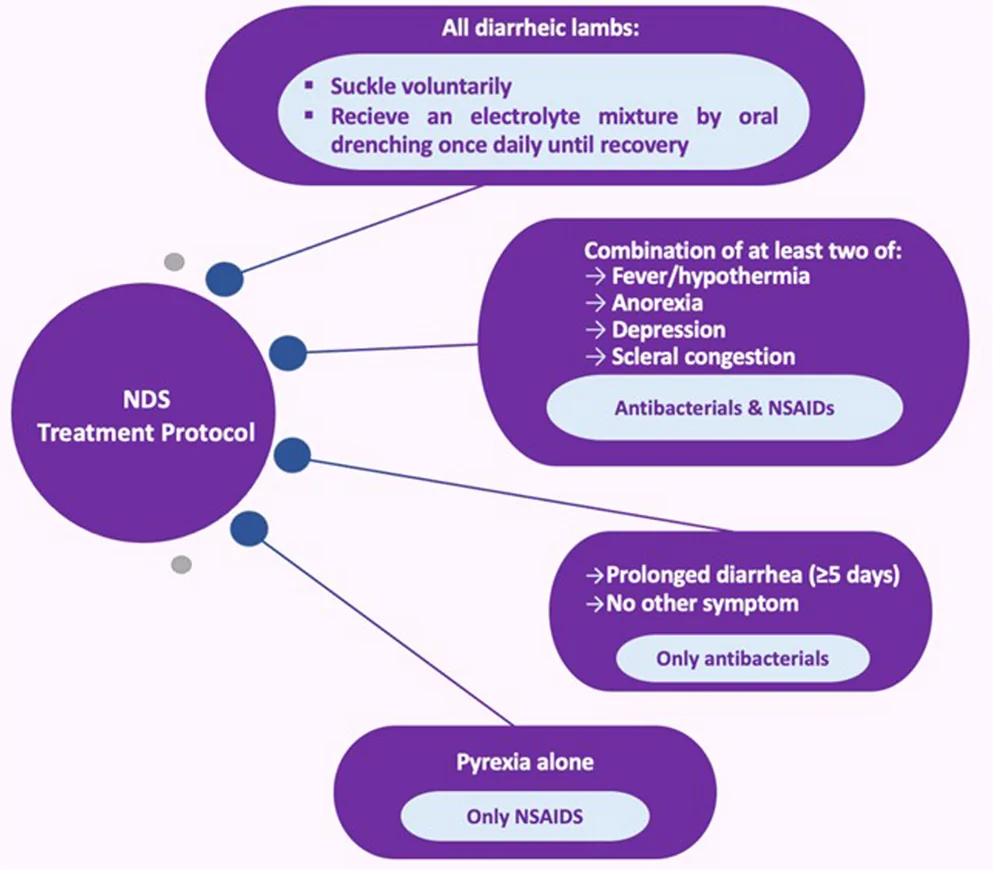
Flow diagram of the evidence-based treatment protocol for neonatal diarrhea syndrome (NDS) in lambs, including oral electrolyte supplementation for all diarrheic lambs, targeted antibacterials and nonsteroid anti-inflammatory drugs (NSAIDs) for ≥ 2 bacteremia signs (pyrexia/hypothermia, anorexia, depression, scleral congestion), antibacterials for prolonged diarrhea (> 5 days), and NSAIDs for pyrexia alone

This prospective case series evaluated a predefined, evidence-based treatment protocol for NDS in lambs. According to the proposed treatment protocol (Fig. 1), all lambs with diarrhea were allowed to suckle voluntarily and receive 150–250 mL of an electrolyte solution as oral drench once daily starting from the first day of diarrhea until recovery, in the presence of suckling reflex. Lambs showing at least two clinical signs compatible with bacteremia (pyrexia or hypothermia, anorexia, depression, and scleral congestion) were to be treated with antibacterials together with nonsteroidal anti-inflammatory drugs (NSAIDs). Lambs presenting only with pyrexia were to be treated initially with NSAIDs alone and lambs with diarrhea persisting for more than five days in the absence of systemic signs were to receive antibacterials alone. Antibacterial selection was based on susceptibility testing.

The protocol was evaluated under field conditions on a commercial Lacaune dairy sheep farm with a documented history of a high incidence of NDS and diarrhea-associated mortality. The study population originated from a group of 95 multiparous ewes that were synchronized to lamb within a defined time frame, allowing uniform monitoring and standardized application of the treatment protocol. These ewes and their lambs were housed together in the same straw-bedded pen throughout the study period and were managed under the same routine farm conditions. During the month preceding the study, neonatal diarrhea was attributed to a mixed infection involving *Cryptosporidium* spp. and Rotavirus A. Farm health records indicated a diarrhea incidence rate of 87%, and a mortality rate of 18%. Liver and kidney samples from two lambs that died during this period were subjected to bacterial identification and susceptibility testing.

The study population comprised lambs born from these ewes over a 60-day period. Of the 133 lambs born during this time, 109 developed diarrhea. Six of them were excluded for medical reasons (2 due to omphalitis and 4 due to pneumonia). Finally, 103 lambs with diarrhea were included in the study. All procedures were performed as part of routine veterinary clinical care.

### Clinical evaluation and laboratory tests – data analysis

Lambs were monitored daily from birth to 20 days of age by the same veterinarian (ATP). Fecal consistency was scored once daily (0 = normal, 1 = intermediate, and 2 = watery feces) (Katsoulos et al. 2017); lambs with scores ≥ 1 were classified as diarrheic. Resolution of diarrhea was defined as a fecal score of 0 maintained for at least 24 h.

During episodes of diarrhea, lambs underwent comprehensive daily clinical examination until recovery. Assessment included rectal temperature, demeanor, scleral vessels, suckling reflex, hydration status, and transabdominal palpation of the abomasum. Dehydration was defined as skin tent ≥ 2 s. Anorexia was defined as the absence of palpable abomasal contents.

All lambs were weighted at birth and blood samples were collected on day 2 of life for the assessment of passive immunity transfer by measuring plasma total protein (TP) concentration and using the cut-off value of ≥ 55 g/l (Gökçe and Ataki̇Şi̇ 2018). Blood samplings also occurred on day 1 of diarrhea and every 48 h thereafter until recovery, with a final sample obtained 24 h post-resolution for the determination of plasma glucose, potassium (K), and sodium (Na) concentrations. These parameters were measured using an automated clinical chemistry analyzer (ADVIA® 1800 Chemistry System; Siemens Healthineers, Germany) with the corresponding commercial diagnostic kits, according to the manufacturer’s instructions.

Fecal samples were collected directly from the rectum on the first day of diarrhea from two lambs per 15 diarrheic cases for etiological investigation. In total, 16 fecal samples were tested for the presence of rotavirus and coronavirus using real-time reverse transcription PCR, for *Cryptosporidium* spp. using the acid-fast Ziehl–Neelsen technique and for *E. coli* via culture onto selective media.

Liver and kidney samples were obtained from all animals that died throughout the study and were subjected to bacterial isolation, identification, and susceptibility testing. Biochemical identification and susceptibility testing were performed using the VITEK® 2 automated system (bioMérieux, France) along with corresponding reagents, and results were interpreted according to Clinical and Laboratory Standards Institute (CLSI) guidelines. The same procedures were applied to bacterial isolates obtained from samples collected prior to study initiation.

The minimum required sample size was determined through a priori power analysis using G*Power (version 3.1.9.7). The study was designed to detect a clinical recovery rate significantly greater than a predefined threshold of 85%, which was established based on reported survival rates for diarrheic lambs under field conditions. Using a one-sample exact binomial test with a target power of 0.80 and a one-sided significance level alpha of 0.05, and assuming an expected recovery rate of 95% the minimum required sample size was calculated to be 78 lambs (actual power 0.81). The enrollment of 103 lambs in this study provided a post-hoc power of 0.89. Statistical analysis of the collected data was performed using JASP (version 0.95.4). The primary clinical outcome—the recovery rate—was evaluated using a one-sample Binomial Test (Exact method) to determine if the observed success rate significantly exceeded the 85% threshold. The 95% confidence intervals (CI) for the recovery and mortality rates were calculated using the Clopper-Pearson (exact) method.

The plasma biochemical data measured (glucose, Na, and K) were analyzed using repeated-measures analysis of variance (ANOVA), with sampling day included as the within-subject factor to evaluate differences across time points. In cases where the assumption of sphericity was violated (assessed via Mauchly’s test), Greenhouse-Geisser corrections were applied. Post-hoc comparisons were performed using the Bonferroni correction to identify specific differences between time points. Statistical significance was set at *P* ≤ 0.05.

### Clinical and laboratory findings

The mean body weight at birth was 3.98 ± 0.8 kg. Adequate passive transfer (TP ≥ 55 g/l) was detected in 94 of 103 lambs (91.3%). The median age at diarrhea onset was 10 days. As is shown in Table 1, pyrexia alone was detected in approximately 27% of diarrheic lambs, while 15% initially presented pyrexia followed in subsequent days by additional systemic signs. Overall, approximately 25% of diarrheic lambs met the predefined clinical criteria for bacteremia, whereas prolonged diarrhea (> 5 days) without systemic signs occurred in 9% of cases.

**Table 1:** Major clinical signs and distribution of the 103 diarrheic lambs at the treatment categories

Treatment category	Major clinical signs	Number of lambs	Percentages (%)
OES	Diarrhea without any other clinical sign	40	38.83
OES, NSAID	Pyrexia alone	28	27.20
OES, AB, NSAID	Pyrexia initially, followed by signs compatible with bacteremia*	15	14.56
	Clinically inferred bacteremia	26	25.24
OES, AB	Prolonged diarrhea (> 5 days) without systemic signs	9	8.73
Total		**103**	**100**

*OES* oral electrolyte solution, *NSAID* nonsteroidal anti-inflammatory drugs, *AB* antibacterials

* Lambs of this category are included in those with Clinically inferred bacteremia

**Fig. 2:**
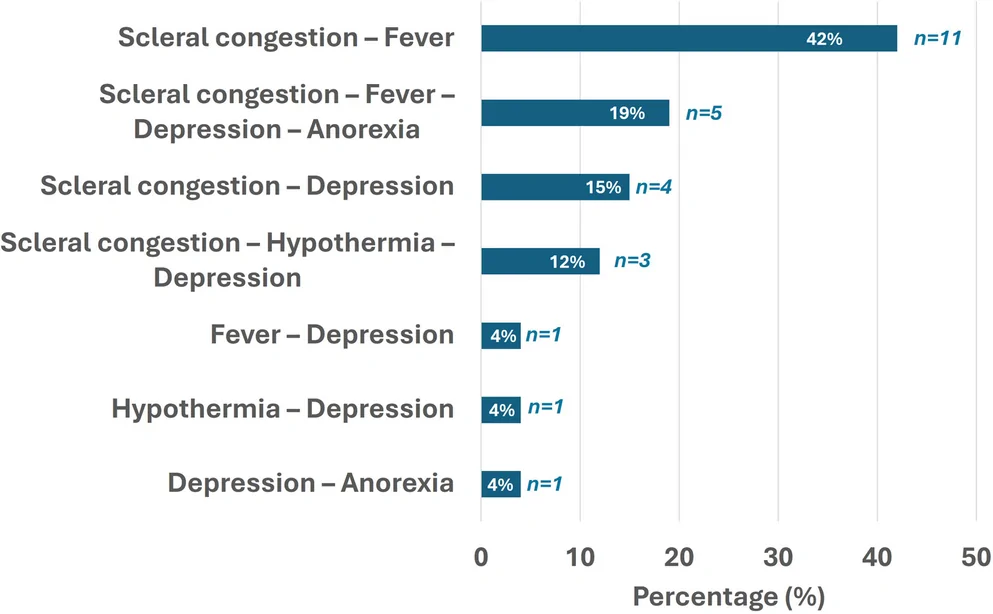
Combinations of clinical signs compatible with bacteremia and their respective frequencies, shown as both number of lambs (n) and percentage (%), observed in diarrheic lambs requiring antibacterial and nonsteroidal anti-inflammatory drug treatment

**Fig. 3:**
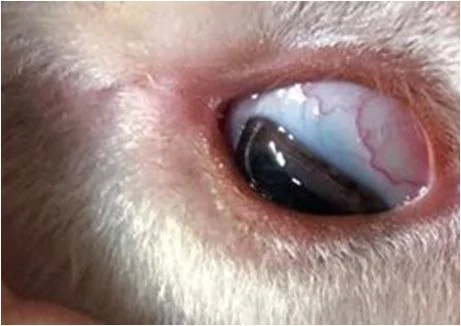
Scleral congestion in a lamb affected by neonatal diarrhea syndrome and concurrent bacteremia

As illustrated in Fig. 2, the most frequent clinical combinations among lambs with signs suggestive of bacteremia were: scleral congestion–pyrexia (*n* = 11; 42%); scleral congestion–pyrexia–depression–anorexia (*n* = 5; 19%); scleral congestion–depression (*n* = 4; 15%); and scleral congestion–hypothermia–depression (*n* = 3; 12%). Less common combinations (*n* = 1; 4% each) included pyrexia–depression, hypothermia–depression, and depression–anorexia. Scleral congestion (Fig. 3) was the predominant clinical finding suggestive of bacteremia, present in 88% of affected lambs, whereas pyrexia was detected in 65% of these cases. Based on the skin turgor test, 20 lambs were classified as dehydrated.

All fecal samples tested were positive for both Rotavirus A and *Cryptosporidium* spp. *E. coli* was the only bacterium isolated from the liver and kidney samples of the two animals sampled before the onset of the study and from those that died during the study period. All isolates were susceptible to all antibacterials tested, except for tetracyclines.

### Treatment and outcome

The treatment protocol was implemented according to the findings of the clinical examination. The oral electrolyte solution (Calfix® Ruminta; Pharmacell, Hellas) was administered via a flexible 6.7 mm diameter orogastric latex tube attached to a 60 mL feeding syringe. When indicated, lambs received a combination of ampicillin and dicloxacillin (Cloxalene Plus®; FATRO S.p.A., Italy) at the dose rate of 10 mg ampicillin + 5 mg dicloxacillin/kg body weight for 3 to 5 days selected based on the susceptibility profile of *E. coli* isolates obtained prior to the study and confirmed during the study period. Meloxicam (Meloxidyl® 20 mg/mL; Ceva Santé Animale, France) was used as the NSAID in the treatment protocol at the dose rate of 0.5 mg/kg body weight, administered once or twice 48 h apart.

The mean duration of diarrhea episodes was 4.0 ± 1.86 days. Three lambs died from *E. coli* bacteremia (mortality rate 2.9%) within two days after the onset of clinical signs compatible with bacteremia. The observed recovery rate of 97.1% (100/103) was significantly higher than the 85% threshold (*P* < 0.001, 95% CI: 91.7–99.4%). Among dehydrated lambs, dehydration lasted one day in 10 cases, two days in 4 cases, and ≥ 3 days in 6 cases. Skin turgor normalized within 24 h post-recovery in 15 lambs and within 72 h in two lambs; three lambs died.

**Fig. 4:**
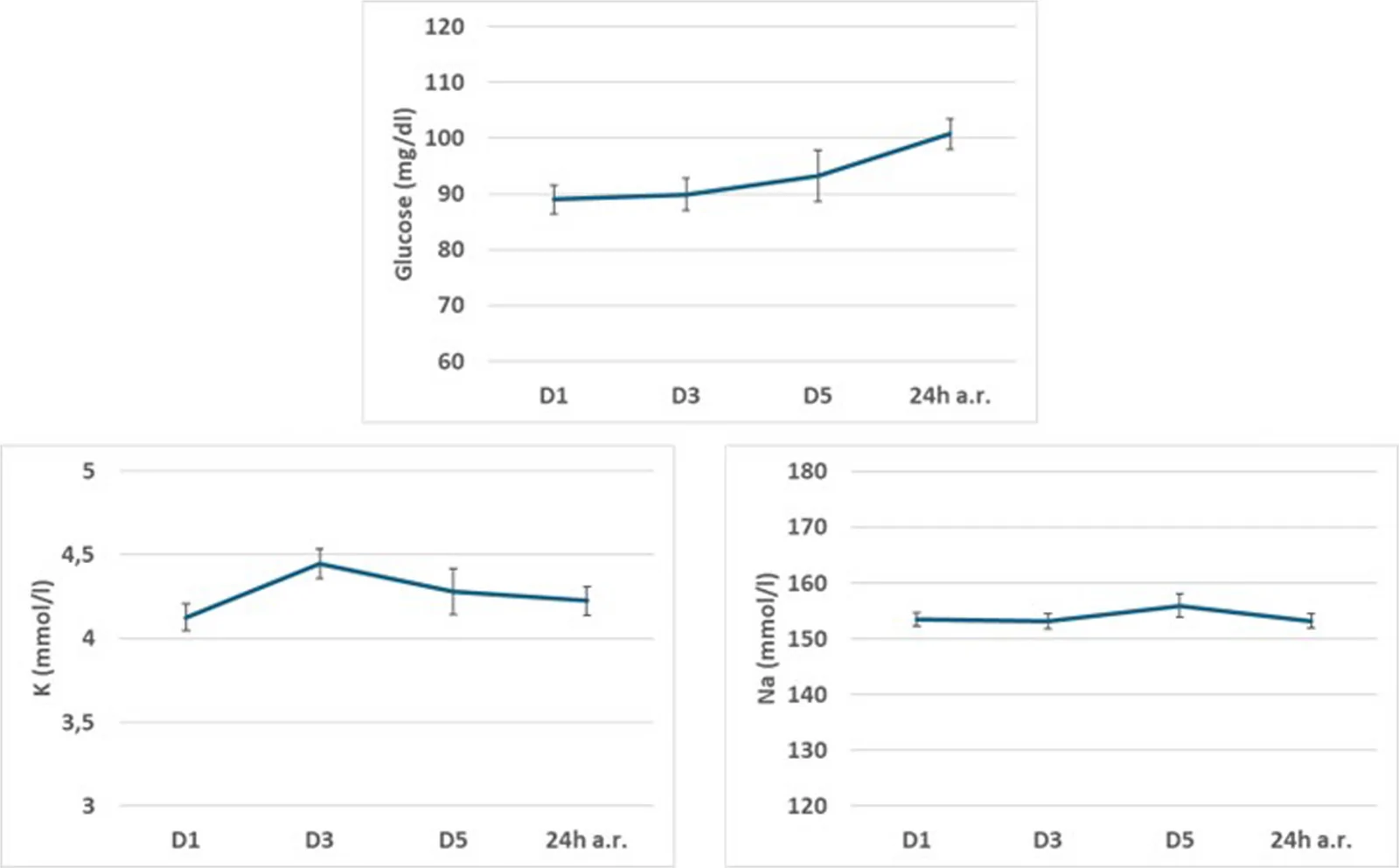
Mean (± SE) plasma concentrations of glucose, potassium (K), and sodium (Na) on day 1 (D1), day 3 (D3), and day 5 (D5) of diarrhea, and 24 h after recovery (24 h a.r.) in lambs with neonatal diarrhea syndrome

As it is shown in Fig. 4 plasma glucose, K, and Na concentrations remained within a narrow range throughout the diarrheic period, and no significant differences were detected between sampling days (*P* > 0.05).

## Discussion and conclusions

This report aimed to develop and evaluate an evidence-based treatment protocol for neonatal diarrhea syndrome in lambs under field conditions. Unlike in calves, there is currently no standardized, evidence-based therapeutic protocol for neonatal lambs, resulting in largely empirical clinical management in sheep production systems. A contemporaneous untreated or blanket-antibiotic comparator group was not included, as the present study was designed as a prospective clinical case series rather than a controlled comparative trial. Withholding supportive treatment from diarrheic neonatal lambs was not considered ethically appropriate, given the risk of dehydration, electrolyte imbalance, energy deficit, and clinical deterioration. Similarly, routine antibacterial administration to all diarrheic lambs from disease onset would not be consistent with antimicrobial stewardship principles, particularly in cases associated primarily with viral and protozoal enteropathogens. Therefore, this prospective field evaluation describes clinical outcomes following implementation of the protocol under commercial farm conditions, while promoting rational therapeutic decision-making through supportive care and targeted antibacterial use based on clinical criteria suggestive of systemic involvement. The absence of a parallel control or alternative-treatment group limits causal inference, and the findings should be interpreted as descriptive field outcomes rather than definitive comparative evidence of superiority. To validate our findings, we utilized a one-sample proportion test against a predefined success threshold of 85%. This benchmark was established based on current literature, where mortality in treated diarrheic lambs typically ranges from 11% to 16% (Sponte et al. 2023; Durgut et al. 2025). The observed recovery rate of 97.1% was significantly higher than this 85% threshold and the lower bound of the 95% confidence interval (91.7%) remained well above the benchmark. These findings support the favorable clinical performance of the proposed protocol under the conditions of the present study. However, given the absence of a parallel control group, they should be interpreted as supportive rather than definitive evidence of efficacy.

Additional limitations include single-farm design, which may limit generalizability, as management practices, pathogen exposure, and antibacterial susceptibility patterns may differ among flocks, breeds, and regions. The study involved a commercial Lacaune dairy flock; therefore, extrapolation to other production systems should be made with caution. Etiological investigation was performed in a subset of diarrheic lambs, and bacteremia in live animals was clinically inferred rather than microbiologically confirmed as no validated gold-standard field test or species-specific clinical scoring system is currently available for neonatal lambs. Therefore, treatment decisions were based on predefined clinical criteria suggestive of systemic bacterial involvement. Clinical assessments and treatment decisions were performed by a single veterinarian and were not blinded, which may have introduced observer bias. However, the use of one experienced examiner ensured consistent case evaluation and uniform application of the protocol, thereby minimizing inter-observer variability.

Direct measurements of colostrum intake, timing of first suckling, and colostrum quality (e.g., Brix refractometry) were not recorded. Under farm conditions, lambs suckled naturally from their dams, making precise quantification impractical. However, lambs were assisted to suckle as soon as they were able to stand, and passive transfer was assessed by serum total protein concentrations on day 2 of life, indicating adequate transfer in the great majority of lambs (> 90%).The proposed protocol follows principles similar to those applied in calves (Berchtold 2009; Constable 2009; Smith 2009; Dawes et al. 2014) and emphasizes the prevention and management of dehydration, electrolyte imbalances, acidosis, and hypoglycemia, as well as the clinical identification of bacteremia to allow targeted antibacterial administration, irrespective of the primary etiological agent of diarrhea. This approach is particularly relevant because no licensed antiviral therapy is currently available for rotaviral diarrhea in lambs, and no approved specific treatment for cryptosporidiosis is available for lambs under our field conditions. Consequently, management of these enteric infections relies mainly on supportive care until spontaneous recovery occurs. In calves, oral electrolyte therapy has been shown to correct these metabolic derangements, restore circulating volume, improve tissue perfusion, and promote recovery regardless of the underlying infectious cause (Miqueo et al. 2018; Wenge-Dangschat et al. 2020; Constable et al. 2021; Bregadioli et al. 2023).

An important distinction between the present protocol and those commonly used in calves is the absence of intravenous fluid therapy. Intravenous fluid administration remains the treatment of choice in severe cases, such as lambs with marked dehydration, recumbency, absence of a suckling reflex, or severe bacteremia. However, its field application is labor-intensive and often impractical, particularly during outbreaks affecting large numbers of animals. In routine flock settings, intravenous therapy may therefore be reserved for individually valuable animals or for cases unresponsive to initial supportive management. Notably, the findings of the present study indicate that when the proposed protocol is implemented promptly and consistently, the number of lambs progressing to such severe clinical states is minimized.

The protocol was effective in managing neonatal diarrhea syndrome under field conditions. The mean duration of diarrhea observed in this study was similar to that reported for uncomplicated rotavirus and *Cryptosporidium* spp. infections in neonatal calves, where diarrheal episodes commonly resolve within a few days with appropriate fluid and electrolyte therapy (Grünberg 2021). Additionally, diarrhea-associated mortality in the present study was very low, at less than 3% and was associated with acute bacteremia. This outcome is much lower than in previous reports. In a recent field trial, a mortality rate of 16% was recorded despite the administration of standard fluid-electrolyte and antibacterial therapy in neonatal diarrheic lambs (Durgut et al. 2025). Higher mortality rates were also recorded in diarrheic lambs receiving either non-specific antibacterial treatment (40–50%) or with etiology-specific treatment (11–12.5%) (Sponte et al. 2023). Other studies recorded mortality rates of 13.8% and 5.1% in diarrheic lambs receiving oxytetracycline plus pepsin and an oral herbal granule, respectively (Li et al. 2015) as well as 13% and 6% in untreated newborn lambs and in those receiving a preventive oral homeopathic product, respectively (Fortuoso et al. 2019).

A key contributor to the success of the protocol, besides the early intervention and close monitoring of the animals, was the continued provision of milk combined with the immediate initiation of oral electrolyte supplementation from the first day of diarrhea, before the development of overt clinical signs of dehydration. The provision of milk supply aligns with recommendations for calves and is also advised for lambs and kids (Diehl 2021). As it is well documented in calves, milk feeding during diarrhea provides an essential energy source and may support intestinal repair, whereas electrolyte solutions alone are insufficient to meet energy requirements (Grünberg 2021). Consistent with this, plasma glucose measurements in the present study demonstrated that this management strategy was effective in maintaining the energy status of lambs throughout diarrheic episodes. Moreover, the early administration of oral electrolyte solutions effectively prevented the development of severe dehydration and electrolyte imbalances. Fewer than one fifth of lambs developed clinical signs of dehydration, and nearly all affected animals had resolved dehydration by the end of the diarrheic period. In addition, plasma Na and K concentrations remained largely stable throughout diarrhea, indicating adequate maintenance of electrolyte homeostasis. The electrolyte solutions were administered via an oroesophageal tube, a practice that is generally discouraged in calves because repeated force-feeding is associated with an increased risk of ruminal acidosis and the development of rumen drinker syndrome (Grünberg 2021). However, no adverse effects attributable to this method of administration were observed in lambs, probably because they remained with their dams and continued to suckle normally throughout the diarrheic period and ruminal drinking is not observed in nursing lambs.

An interesting observation was that pyrexia in the absence of other clinical signs suggestive of bacteremia, persisting for 24–48 h, was detected in approximately 27% of diarrheic lambs and occurred either at the onset of diarrhea or one day before. The origin of this transient pyrexia remains unclear; however, it may be associated with rotaviral infection, as in children, rotavirus disease typically begins with the acute onset of fever and vomiting, followed one to two days later by diarrhea (Parashar et al. 2013). Consequently, the presence of pyrexia alone should not be interpreted as indicative of bacteremia, nor does it justify the administration of antibacterial agents or predict poorer clinical outcomes. In such cases, the administration of NSAIDs is recommended in our protocol to mitigate the effects of pyrexia, particularly its negative impact on milk intake, and to support animal welfare. Moreover, NSAID administration in these lambs was considered safe, as oral electrolyte supplementation had already been initiated, thereby minimizing the risk of dehydration. Apart from these animals, an additional 15% of diarrheic lambs initially presented with pyrexia as the sole clinical finding but subsequently developed signs compatible with bacteremia within a few days. This observation highlights the importance of close clinical monitoring of lambs presenting with pyrexia, as it may precede the onset of systemic infection.

To avoid misinterpretation of pyrexia alone as an indicator of potential bacteremia and to prevent unnecessary antibacterial use, the study protocol required the presence of at least two clinical signs compatible with bacteremia to justify antibacterial administration in diarrheic lambs. Although, to our knowledge, no validated clinical scoring system for the diagnosis of bacteremia exists specifically for neonatal lambs, similar combinations of clinical signs have been incorporated into clinical sepsis scoring systems used to identify suspected bacteremia or septicemia in neonatal calves (Fecteau et al. 1997a). Based on the application of these criteria, evidence compatible with bacteremia was identified in 25% of the diarrheic lambs in the present study. This proportion is comparable to the prevalence of bacteremia typically reported in diarrheic calves, which is approximately 30% (Fecteau et al. 1997b; Lofstedt et al. 1999), and is generally attributed to secondary systemic infection with *E. coli* (Constable 2004).

Among lambs exhibiting clinical signs compatible with bacteremia, scleral congestion was the most prevalent sign, as was observed in 88% of affected lambs. This is a particularly important observation, as scleral congestion is a readily recognizable clinical sign that can be reliably identified by farmers following appropriate training, thereby facilitating early detection of systemic illness in field. Similarly, scleral congestion has been reported as the most common clinical sign in diarrheic calves with bacteremia (Aldridge et al. 1993; Fecteau et al. 2009; Dawes et al. 2014). In contrast, and in agreement with observations in calves (Aldridge et al. 1993; Fecteau et al. 2009), pyrexia was present in approximately two third of the lambs with signs compatible with bacteremia, indicating that fever is not a consistent or reliable clinical indicator of bacteremia in neonatal lambs.

According to the protocol, antibacterial therapy was administered not only to lambs exhibiting clinical signs compatible with bacteremia but also to animals with prolonged diarrhea. This usually concerns a relatively small number of animals, which in our case was about 9%. The > 5-day threshold was established as a pragmatic clinical criterion rather than one specifically validated in lambs, since evidence-based data defining prolonged diarrhea in this species is currently lacking. It was selected because diarrhea persisting beyond this period was considered clinically relevant in neonatal lambs and potentially associated with continued fluid loss, impaired nutritional status, delayed mucosal recovery, secondary bacterial overgrowth, and reduced welfare. Although persistent diarrhea does not necessarily indicate systemic bacterial infection, differentiation between normal post-infectious recovery, enteric dysbiosis, and cases requiring further treatment is often not feasible under field conditions. Therefore, antibacterial administration was used as a practical intervention in unresolved cases in order to support intestinal recovery and prevent further complications.

Overall, antibacterial agents were administered to 34% of diarrheic lambs, demonstrating that most cases can be managed without antibacterial therapy and that antibacterial use was justified in approximately one third of lambs with diarrhea. This finding underscores the importance of a targeted treatment approach, as indiscriminate antibacterial administration not only increases the risk of antibacterial resistance but might also adversely affect clinical outcomes. Indeed, in diarrheic calves, the unjustified use of antibacterials has been associated with a prolongation of diarrhea duration by up to 70%, likely due to disruption of normal intestinal microbiota and delayed mucosal recovery (Berge et al. 2009). These observations further support the use of strict clinical criteria to guide antibacterial administration in neonatal diarrhea, thereby promoting antimicrobial stewardship and supporting optimal animal recovery.

The evidence-based treatment protocol developed in this study, based on the early intervention and close monitoring of lambs proved effective for managing NDS in lambs in field by supporting the animals during the disease process, preventing complications and improving the likelihood of recovery. Early and consistent oral electrolyte therapy was essential for maintaining hydration, preserving metabolic balance, and improving clinical outcomes. The protocol enabled timely identification and treatment of systemic involvement using clear clinical criteria, while restricting antibacterial therapy to cases with convincing clinical evidence of bacteremia and in cases of prolonged diarrhea. Notably, pyrexia alone was not a reliable trigger for antibacterial use. By prioritizing supportive care and targeted, judicious antibacterial administration, this approach reduces unnecessary antibacterial exposure, mitigates resistance risks, and offers a pragmatic, ethically sound framework for the responsible management of NDS in lambs. Of course, further studies in different flocks and production systems are warranted to confirm the applicability of the proposed protocol and to further evaluate its performance under varying field conditions.

## Data Availability

Not applicable.
